# Enlargement of the Lower End of the Femur

**Published:** 1909-05-29

**Authors:** 


### Surgery.

### ENLARGEMENT OF THE LOWER END OF THE FEMUR.

The differential diagnosis of the conditions which cause enlargement of the lower end of the femur, particularly when the condition is a chronic one, may be most perplexing.

Even in acute disease the recognition of the cause is not always an easy matter, and cases in which osteomyelitis of the femur has been mistaken for and treated as rheumatism of the knee-joint must have come within the experience of most of our readers. But if the clinical aspect of the case is carefully considered, there is really little excuse for such an error. To start with, osteomyelitis generally occurs in children who are recovering from one of the acute exanthemata by which their resistance to a hæmic infection has been markedly diminished. There may or may not be a history of a slight injury to the limb.

The disease runs an acute course with pronounced constitutional symptoms, so that the child looks very ill and complains of great pain in the limb. There is also continued pyrexia, the temperature often remaining persistently high, and occasionally reaching 105° F. Rigors are a not infrequent feature. In acute rheumatism there are constitutional symptoms of a similar order, though not so severe, and the swelling of the joint is transient. In a chronic rheumatic joint the constitutional symptoms are absent. But a careful examination of the limb will reveal greater discrepancies. In osteomyelitis the greatest swelling in the circumference of the limb occurs not at the level of the joint, but above it. The joint itself is unaffected, and there is no evidence of free fluid in it, although movements may be limited on account of pain. In rheumatism, acute or chronic, the swelling is in the joint itself; movement causes great pain, and the normal anatomy of the joint is modified by fluid in the synovial membrane.

Unfortunately, salicylate in some form is often given in doubtful cases. Now, apart from its specific action on rheumatic affections, salicylates have an antipyretic effect, so that a false sense of security may result, if it is argued that a fall in the temperature denotes that the case is certainly rheumatic in origin. One other acute condition may give rise to great difficulty in diagnosis: this is infantile scurvy. It is a disorder of childhood which is caused by injudicious diet. It is characterised by constitutional symptoms, anæmia, pyrexia, and anorexia; but its distinguishing feature is that it causes hæmorrhages under the skin, from mucous membranes, and under the periosteum. When the subperiosteal hæmorrhage occurs in the lower end of the femur it causes an enlargement of the lower end of the bone, which is very tender; and this may be confused with an osteomyelitis. But the swelling is usually confined to one part of the circumference of the bone, and does not extend all round, as in osteomyelitis, and this fact, combined with the presence of petechiæ and hæmorrhage from the gums or mucous membrane, should be sufficient to put one on one's guard.

The problem presented by chronic swellings is one of greater difficulty. Roughly speaking, the question here resolves itself into this: is the swelling due to a chronic inflammatory process, or is it a new growth? A sclerosing periostitis leading to a general enlargement of the bone is less common in the femur than it is in the tibia. This is probably due to the fact that the femur is well covered by muscle, while the tibia occupies an exposed position; an injury is a common factor in the causation of this condition. On the other hand, central abscess or necrosis with distension of the shaft of the bone is more often found in the femur, and it is these cases that are so difficult to differentiate from neoplasm.

A positive history of past syphilitic infection or the presence of a tuberculous focus in some other part of the body will, of course, be helpful, and the presumption then is that the enlargement of the femur is due to the same cause. Apart from this, little help is usually obtained. The swelling comes on gradually, and no cause can be assigned for its commencement. It may or may not be associated with pain of a dull boring character, which is sometimes sufficiently intense to keep the patient awake at night, or to cause him to limp during the day. Movement at the knee-joint may be limited; but this comes on later, and is secondary to the overgrowth of the lower end of the femur, and is not caused by changes in the joint. There may be pyrexia in sarcoma just as in inflammatory conditions.

The femoral glands may be enlarged in either condition. It is commonly said that sarcoma does not cause enlargement of the lymphatic glands which drain the site of the lesion, except when the growth involves the tonsil, testis, or thyroid. But in actual practice it will be found that such a rule is more honoured in the breach than in the observance; in fact, in the last three cases of sarcoma starting in bone or periosteum which have come under the writer's observation, the primary lymphatic glands were invaded by sarcoma cells in all of them.

The one point on which reliance may be placed is this: a periosteal sarcoma forms a hard discrete mass which is well defined, and its upper border ends sharply. An inflammatory swelling ends gradually and shelves off into the normal bone. Moreover, it is generally tender on percussion; this is characteristic of all inflammatory bony swellings. If the case can be watched, iodide of potassium should be given, and in a syphilitic case improvement will begin to show itself; or again, a central tuberculous focus may make its way to the surface and declare itself as a definite abscess.

A skiagram of the swelling should always be taken, since valuable evidence may be thus obtained, especially if the services of an expert radiographer can be obtained. But it is not easy, without a large experience, to distinguish between a central sequestrum surrounded by involucrum on the one hand, and the shaft of a femur embedded in a periosteal sarcoma on the other. But the diagnosis may remain in doubt, even after all ordinary methods of examination have been tried. In such a case an exploratory incision is necessary.

